# Granzyme B Inhibits Vaccinia Virus Production through Proteolytic Cleavage of Eukaryotic Initiation Factor 4 Gamma 3

**DOI:** 10.1371/journal.ppat.1002447

**Published:** 2011-12-15

**Authors:** Marcelo Marcet-Palacios, Brenda Lee Duggan, Irene Shostak, Michele Barry, Tracy Geskes, John A. Wilkins, Akiko Yanagiya, Nahum Sonenberg, R. Chris Bleackley

**Affiliations:** 1 University of Alberta, Department of Biochemistry, Edmonton, Alberta, Canada; 2 University of Alberta, Department of Medical Microbiology and Immunology, Edmonton, Alberta, Canada; 3 University of Manitoba, Manitoba Centre for Proteomics & Systems Biology, Winnipeg, Manitoba, Canada; 4 McGill University, Department of Biochemistry, Montreal, Quebec, Canada; Fox Chase Cancer Center, United States of America

## Abstract

Cytotoxic T lymphocytes (CTLs) are the major killer of virus-infected cells. Granzyme B (GrB) from CTLs induces apoptosis in target cells by cleavage and activation of substrates like caspase-3 and Bid. However, while undergoing apoptosis, cells are still capable of producing infectious viruses unless a mechanism exists to specifically inhibit viral production. Using proteomic approaches, we identified a novel GrB target that plays a major role in protein synthesis: eukaryotic initiation factor 4 gamma 3 (eIF4G3). We hypothesized a novel role for GrB in translation of viral proteins by targeting eIF4G3, and showed that GrB cleaves eIF4G3 specifically at the IESD^1408^S sequence. Both GrB and human CTL treatment resulted in degradation of eIF4G3 and reduced rates of translation. When Jurkat cells infected with vaccinia virus were treated with GrB, there was a halt in viral protein synthesis and a decrease in production of infectious new virions. The GrB-induced inhibition of viral translation was independent of the activation of caspases, as inhibition of protein synthesis still occurred with addition of the pan-caspase inhibitor zVAD-fmk. This demonstrated for the first time that GrB prevents the production of infectious vaccinia virus by targeting the host translational machinery.

## Author Summary

Lymphocytes, a type of white blood cell, are the major killer of virus-infected cells. Lymphocytes secrete proteins like granzyme B that are responsible for the destruction of the virus-infected host cell. However, killing an infected cell through this pathway may take several hours, thus allowing viral replication to occur while the cell is in the process of dying. In this study, we identified a new role of granzyme B in preventing viral replication during the killing process. We found that granzyme B disables the ability of the host cell to make new proteins, including viral proteins of infected cells. Thus, granzyme B is able to halt the production of new viruses by inhibiting protein production.

## Introduction

One major strategy of the host to survive the attack of viruses is to induce apoptosis in infected host cells. Cytotoxic T-lymphocytes (CTLs) play an important role in the apoptosis pathway, which activates a family of cytosolic proteins called caspases in target cells. When caspases are activated, they execute the key reactions that drive target cells to their demise. Activation of initiator caspases such as caspase-8 and 10 results in direct activation of the apoptosis executioner caspases like caspase-3 [1]. Caspase-8 and 10 also signal through the mitochondrial pathway by activating a protein called “BH3 interacting domain death agonist” (Bid) [2], [3], resulting in the release of cytochrome c (cyt c). Soluble cyt c also mediates the activation of the executioner caspases [1]. Thus, there is cooperation between the mitochondrial pathway and the caspase system. Active caspase-3 cleaves ICAD (inhibitor of caspase-activated deoxyribonuclease), with subsequent release of CAD and DNA degradation. Other substrates of executioner caspases include cytoskeletal and nuclear skeletal components like fodrin and lamin A, which result in cell shrinkage [1].

The mechanism by which CTLs activate the caspase cascade system has been an active area of research. We now know that electron dense granules found in CTLs carry cytolytic factors that trigger apoptosis in target cells. Granules polarize toward the immune synapse as the membranes of the CTL and target cell make contact. Cytolytic factors in the granules are then delivered to the target cell to induce cell death. Two of the first proteins to be isolated from these granules were perforin [4], [5] and granzyme B (GrB) [6]. Although purified perforin readily lyses cell membranes, perforin alone is not able to initiate DNA fragmentation in the same way as treatment with CTLs [7]. Combined treatment with perforin and GrB reproduces the effects of CTL treatment [8], by inducing both membrane damage and DNA fragmentation. GrB is a serine protease with an unusual substrate specificity, cleaving proteins at aspartic acid residues [9]. GrB is initially synthesized as an inactive zymogen that is activated by the removal of two amino acids at the amino terminus [10]. In the current model of CTL-mediated killing, perforin plays a role in granting GrB access to the cytosol of target cells [11]. Through proteolytic cleavage, GrB activates cytosolic substrates such as caspases [12], [13] and Bid, independent of caspase-8 [14]. Proteolytic activation of Bid results in heterodimerization with Bax (B-cell CLL/lymphoma 2 (Bcl2) associated X protein) and the subsequent recruitment of the Bid/Bax complex to the mitochondria. The Bid/Bax complex promotes mitochondrial membrane depolarization [15] and the release of cyt c and SMAC (second mitochondria-derived activator of caspase). SMAC binds and blocks the actions of caspase inhibitors, namely the inhibitor of apoptosis proteins (IAPs) [16]. Thus, GrB is a powerful pro-apoptotic factor that activates executioner caspases directly and through the mitochondrial pathway.

A cell infected with virus becomes a target for destruction by CTLs via the GrB pathway. However, while under attack, the production of infectious virus can still occur and be released from the dying cell. Thus, viral infection of the host would ensue regardless of cell death. It would make sense if CTLs had a strategy to stop the virus in its tracks. In addition, to evade host defenses, viruses have evolved mechanisms to inhibit the caspase-cascade system. For example, vaccinia virus (VV) expresses the cytokine response modifier A (CrmA), which is a serine protease inhibitor or serpin. CrmA is a strong inhibitor of caspase-1, 3, 6 and 8 [1], thereby acting to delay the process of apoptosis. Thus, it is possible for viruses such as VV to use dying cells for viral replication. The question arises as to whether CTLs have strategies to deal with this ongoing production of infectious virus. Recent literature reveals new roles of human granzymes that extend beyond apoptosis. Two of these emerging roles involve activation of the inflammatory system and direct antiviral functions that are independent of cell death. The ability of granzymes to induce and process pro-inflammatory cytokines demonstrated that granzymes activate the inflammatory system [17], [18]. Granzymes also have the potential to directly impair viral replication with an increasing list of host and viral substrates involved in the replication process of viruses [18]. GrH can cleave and inactivate host and viral substrates to prevent viral replication. Romero *et al*. demonstrated that GrH-mediated cleavage of the human protein La decreased hepatitis C virus IRES-mediated translation [19]. One of La's roles is to stimulate IRES-mediated translation, a mechanism used by many viruses to take control of the host translational machinery. In a different study, Andrade *et al.* identified adenovirus protein 100K as a novel GrH substrate [20]. 100K is important in the assembly of the virus capsid and also functions as a GrB inhibitor. Thus, the authors uncovered a role in which GrH inactivated adenovirus replication in a pathway that could concomitantly de-repress GrB activity. It seems clear that granzymes have evolved unique antiviral strategies that are independent of their well-characterized cytolytic roles. In this study, we propose a novel mechanism for GrB to directly inhibit the production of VV proteins and thus, inhibit viral replication.

In search for novel GrB substrates, we found eukaryotic initiation factor 4 gamma 3 (eIF4G3), a protein critical for initiation of protein translation. In humans, translation is largely regulated by eukaryotic translation initiation factors (eIF). Detailed reviews of the events involved in this process have been published earlier [21], [22]. Briefly, a newly transcribed host mRNA is transported to the cytosol in its linear conformation. Prior to the initiation of translation, a multi-subunit complex called eIF4F needs to be formed. Subunits within the complex have important roles. First, to flank the mRNA, the eIF4E subunit binds to the 5′ end of the mRNA, while the poly(A) tail binding protein (PABP) binds to the 3′ end. Following this, the large scaffold subunit eIF4G3 circularizes the mRNA molecule by binding to both PABP and eIF4E. The eIF4G3 protein is a central player in the initiation of translation since it also contains binding domains for mRNA, eIF4A, and eIF3 (itself a multi-subunit factor that recruits the ribosome). It is not surprising that many viruses interfere with the function of the eIF4F complex to gain control of translation. Some viruses cleave eIF4G3 to halt host protein translation while other viruses use eIF4G3 to make viral proteins. For example, rhinoviruses and enteroviruses shut off host protein synthesis by cleavage of eIF4G3, resulting in inhibition of cap-dependent translation [23]. Cleavage of eIF4G3 is carried out by the viral-encoded protease 2A^pro^. In contrast, most viruses including poxviruses, adenoviruses, papillomaviruses, polyomaviruses, herpesviruses, and asfarviruses do not cleave eIF4G3, but instead, take full advantage of the eIF4F complex [24]. VV, a double-stranded DNA virus of the poxvirus family, cannot replicate in the absence of eIF4G3 [23], [25], [26]. VV translates its RNA in a cap-dependent manner, increases eIF4F complex levels and enhances PABP binding [24], [27]. Thus, we used VV as a model system to study the role of eIF4G3 degradation by GrB in blocking viral infection.

We hypothesized that in addition to pro-apoptotic roles, GrB also targets the translational machinery by cleaving eIF4G3. In the absence of a functional eIF4G3, translation initiation would be arrested. In this study, we demonstrate that eIF4G3 cleavage by GrB is the critical event that prevents VV protein synthesis and reduces subsequent production of infectious VV particles.

## Materials and Methods

### Cells and viruses

Jurkat cells were obtained from American Type Culture Collection (ATCC). Jurkat cells were passaged approximately every 2 days when cell density reached 1×10^6^ cells/ml to bring cell density back to 1×10^5^ cells/ml. Cells were grown in RPMI supplemented with 10% fetal bovine serum (FBS), 0.06 mg/ml penicillin G, and 0.01 mg/ml streptomycin sulfate. Cell number was then adjusted prior to each experiment as needed for each method below. Human CTL line was generated as previously described [28] and maintained in RHFM media containing 90 U of interleukin 2 (Chiron) per ml. Treatment of Jurkat cells with CTL generated an allo-response. African “Buffalo Green” Monkey Kidney (BGMK) cells and Vaccinia Virus (VV) were obtained from ATCC and cultured as previously described [29]. Replication-deficient adenovirus (AD) (type 5) was a generous gift from Jack Gauldie, McMaster University.

### Inhibition of GrB activity

The GrB inhibitor L-038587-00Y001 (GrB inh) was a kind gift from Dr. Nancy A. Thornberry (Willoughby et al., 2002). The inhibitor was incubated with GrB for 30 min at a concentration of 10 µM at 37°C prior to treating cells with GrB/AD.

### AD-mediated internalization of granzyme B (GrB)

A non-replicating strain of AD was used as previously described [30] to internalize GrB into cells. AD was used at 1 plaque forming unit (PFU)/cell in all experiments involving purified GrB-mediated killing of cells.

### GrB enzymatic assay

GrB activity was measured in a colorimetric assay [31]. Substrate degradation of Ac-IEPD-pNA (Kaiya Biomedical, Seattle WA) was monitored at A_405_. GrB-mediated hydrolysis of Ac-IEPD-pNA was inhibited in the presence of purified serpina3n (0.6 to 0.0001 mg/mL) or GrB inh.

### Plaque assay

In these experiments, 2.5×10^6^ Jurkat cells were infected with VV at a multiplicity of infection (MOI) of 10. Unless otherwise specified, GrB concentration was 1 µg/ml (∼30 nM). AD concentration was always the same at 10 PFU/cell. All treatments that contained GrB also contained AD and zVAD-fmk (50 µM, 30 min prior to GrB/AD/VV). Following infection with VV (6 hr), cells were treated with GrB/AD for 1 hr, washed 3 times in PBS and then lysed. To estimate the number of VV present in the cell lysates, plaque assays were performed. BGMK were grown in monolayers and infected with the cell lysates as previously described [29].

### Cleavage by GrB and mass spectrometry

Jurkat cells were co-treated for 30 min at 37°C with GrB at 1 µg/ml and AD at 10 PFU/cell. Following cell fractionation, cytosolic fractions were analyzed by 2-Dimensional electrophoresis and silver staining. This technique was performed as previously described with some modifications [32]. Cells were centrifuged for 1 min at 18,000 g and the pellet was resuspended in total lysis buffer and vortexed for 1 min. Proteins were purified and desalted using the ReadyPrep 2-D Cleanup kit (Bio-Rad, ON, Canada) and resuspended in 120 µl of rehydration buffer as provided by Bio-Rad. Samples containing 120 µg of protein were electrofocused on Immobiline DryStrip pH 3–17, 7 cm IPG strips (GE Healthcare, NJ, USA) using the IPGphro isoelectric focuser (Pharmacia Biotech). Strips were equilibrated in ReadyPrep Equilibration buffers I and II (Bio-Rad) for 30 min in each buffer. Second dimension was run in Ready Gels 8–16% Tris-HCl, IPG COMB (Bio-Rad). Following electrophoresis, gels were fixed and silver stained using the silver staining plus kit (Bio-Rad). Mass spectrometry analysis was conducted as previously described [33]. Jurkat cell extracts were also tested by treating with inactive and active GrB, and ran in 1-Dimensional gels. Gel slices were analyzed by mass spectrometry at the Manitoba Centre for Proteomics and Systems Biology.

### [^3^H]-Thymidine release assay

GrB and CTL-mediated cytolytic activity was measured using methods that have been previously described [14], . 3×10^5^ cells/ml of Jurkat cells were labeled with 40 µCi ^3^H-thyamidine for 20 hr prior to the experiment. Cells were treated with GrB at 1 µg/ml and AD at 10 PFU/cell. Human CTL-mediated killing was conducted as above with the following modifications. Jurkat cells were set at 1×10^6^ cell/ml from 16 ml of total cell suspension. Cells were treated with a human CTL line [28] at an effector to target ratio of 5 to 1. Jurkat cells were killed by either GrB treatment or CD8-mediated killing, then kept in the incubator for the duration of the experiment. At each time point, triplicates of 200 µl from each tube were collected. A total of 20 time points were collected, once every 15 min from 0 min to 300 min. Immediately after collection, 200 µl/tube of lysis buffer (1% Triton x100 in TE buffer) was added and vortexed for 1 min. Cells were then centrifuged for 10 min at 18,000 g in a Microfuge 22R (Beckman Coulter) at 4°C. From the supernatant, 200 µl was collected and mixed with 5 ml of scintillation fluid. Each individual tube was counted for 2 min. Total ^3^H (100% Detectable DNA) was measured by collecting five-50 µl (quintuplicates) samples from each tube above. To each of these five tubes, 400 µl of total lysis buffer (2% SDS) was added and vortexed for 1 min. 5 ml of scintillation fluid was added and samples were counted for 2 min each. Cytolytic activity was measured by calculating the percentage of specific [^3^H]-thymidine release as follows: 100 x [(sample cmp – spontaneous cmp)/(total cpm – spontaneous cpm)]. The pan-caspase inhibitor Z-Val-Ala-Asp(OMe)-CH_2_F (zVAD-fmk) was used to assess the role of caspases (Kamiya Biomedical Company, WA, USA). In these experiments, cells were incubated with 50 µM zVAD-fmk for 30 min prior to GrB treatment.

### FITC-annexin V binding

Inversion of phosphatidylserine on apoptotic plasma membrane was determined by FITC-annexin V binding and flow cytometry (Kamiya) [35].

### Evaluation of protein synthesis

Jurkat cells were pulse-labeled with 33 µCi [^35^S]methionine (PerkinElmer) for 1 hour at a density of 3×10^5^ cells/ml. Following treatment, cells were centrifuged for 1 min at 18,000 g. Proteins were recovered through acetone purification using the ReadyPrep 2-D Cleanup kit. To each protein pellet, we added 400 µl of total lysis buffer (2% SDS) and vortexed for 1 min. 5 ml of scintillation fluid was added and samples were counted for 1 min each. Protein concentration was determined using the BCA assay (Pierce). The amount of total translated protein was estimated through the incorporation of Met^S35^ per mg of total protein (% Translated Protein). The highest count per minute (CPM) per mg of protein for each experiment was considered 100% translated protein. % Translated Protein was calculated from this value and plotted over time.

### SDS-PAGE

10% acrylamide gels were used. Electrophoresis was run in a mini protean 3 cell (Bio-Rad) connected to a Power Pac 1000 (Bio-Rad).

Quantitative PCR (qPCR): This assay was performed as previously described [36]. β-actin primers were: forward 5′-CGA GAC CAC CTT CAA CTC CAT C-3′ and reverse 5′-GCG GTG GAT GGA GGG-3′.

### In vitro transcription/translation (IVTT) and in vitro translation (IVT)

The TNT quick-coupled transcription/translation kit was used (Promega, WI, USA). This kit is a rabbit transcription/translation system. The translational machinery is thus Rabbit and the transcriptional machinery is added as a supplement to the rabbit reticulocyte lysate and is of bacteriophage origin. Master mix aliquots were thawed by hand warming and placed on ice. Individual reactions were prepared by combining 40 µl of master mix with 0.02 µg/µl of template DNA, 0.04 mM [^35^S] Methionine (Met^S35^) and water up to a total of 50 µl. Tubes were incubated at 30°C for 90 min. To perform IVT, the above method was modified by substituting the template DNA with purified 10 µg/µl mRNA, in the presence of 0.02 U/µl of DNase. To control for translation, the reaction was arrested by adding 0.01 U/µl of RNase. Radiolabeled proteins were used for *in vitro* proteolysis studies, or further purified by affinity chromatography. Visualization was performed by SDS-PAGE followed by either silver staining or autoradiography of the dried gels.

### In vitro proteolysis

IVTT or IVT reaction products were put on ice immediately after synthesis. GrB was diluted in PBS to yield a working concentration of 30 nM unless otherwise specified. Proteolytic degradation by GrB was carried out in a water bath for 30 min at 37°C and terminated by adding 6 µl of sample solubilizing buffer (SSB) (0.5 M Tris-HCl, 16% glycerol, 3.2% SDS, 0.8% β-mercaptoethanol, 10 µg/ml bromophenol blue). The resulting proteolytic products were visualized by silver staining and autoradiography (Biomax MR film, Kodak, NY, USA) following SDS-PAGE.

### Mutagenesis

Point mutations were carried out using the Quikchange site-directed mutagenesis kit (Stratagene, CA, USA). The vector pcDNA3 containing eIF4G3 was provided by the lab of Dr. Nahum Sonenberg (McGill University, Canada). Oligonucleotide primers (Forward 5′-GTT GGA CTT CAT AGA GTC TGC CAG TCC CTG TTC CTC TGA AG-3′ and Reverse 5′-CTT CAG AGG AAC AGG GAC TGG CAG ACT CTA TGA AGT CCA AC-3′) were designed to point-mutate aspartate^1408^ (D^1408^) to alanine. Mutated plasmids were transformed into competent DH5α cells (Invitrogen, ON, Canada). Mutations were confirmed by sequencing the inserts. Successful colonies were purified using the EndoFree kit (Qiagen, ON, Canada).

### Metabolic labeling of VV-infected cells

This technique was carried out as previously described with the following modifications [37]. Jurkat cells were mock infected or infected with VV at a MOI of 10 for 0 to 18 hr. 1 hr prior to termination of infection, Jurkat cells were treated with GrB at 1 µg/ml and AD at 10 PFU/cell. Cells were then starved for 30 min and pulse-labeled with 33 µCi [^35^S]methionine and [^35^S]cysteine (PerkinElmer) for 1 hour. Samples were analyzed by SDS-PAGE followed by autoradiography.

### Stable transfection

Human Jurkat cells were transfected with pcDNA3-EIF4G3 and pcDNA3-EIF4G3 asp/ala mutant using the Amaxa® cell line Nucleofector® (Lonza, Cologne Germany). Briefly, 1.5×10^6^ cells were transfected with 2 µg Pvu I linearized plasmids using the Amaxa® Kit V and the program X-05. The transfected cells were then transferred to 1 ml pre-warmed RHFM media in a 12 well plate. After a 24 hr incubation period in a humidified 37°C/5% CO_2_ incubator stably transfected cells were selected using 1 mg/ml G418 (Gibco BRL Life Technologies inc.) and cloned by the limiting dilution method. Four mutant clones and 12 wild type clones were selected and maintained in 0.8 mg/ml G418. Two clones from this group (mutant #4 and wild type #12) were chosen for further analysis. To confirm the integration of the plasmid DNA into the host chromosomes, genomic DNA was extracted from the two clones and a PCR fragment was generated using the 5′ eIF4G3 primer and the 3′ bovine growth hormone primer. The resulting PCR fragment was purified and sequenced.

### Affinity chromatography

This technique was conducted using the ProFound Mammalian HA-Tag IP/Co-IP kit (Pierce, IL, USA). HA-Tagged eIF4G3 and the mutagenized clone eIF4G3^Δ^ were generated through IVTT in the presence of Met^S35^. Following a total of 12 independent IVTT reactions for each clone, volumes were combined and loaded into the column. A total of 18 µl of anti-HA agarose slurry (30 µg anti-HA antibody) was then added to the sample and incubated for 16 hr at 4°C. Columns were then washed 3 times and the HA-tagged proteins were eluted into a 50 µl final volume. Protein concentrations were assessed by BCA assay (Pierce). Purification efficiency and protein identity was assessed respectively by autoradiography and Western blot analysis.

### Western blot analysis

Samples were subjected to 10% SDS-PAGE and proteins were identified using 1 µg/ml polyclonal anti-eIF4G3 antibody (Abcam, MA, USA).

### Densitometric analysis

Blots or films were scanned at 500 dots per inch in the Agfg Arcus II scanner using Adobe Photoshop. The scanned images were saved as grey .tif files. Densitometric analysis was performed with the SigmaGel software. For each reading, a measurement was taken of the background and subtracted from the reading generated from the bands. For each film or blot, measurements were performed 3 times and the average was calculated. A number in arbitrary units was used to calculate the percent of expression.

### Statistical analysis

Results are means ± S.E. of at least three independent experiments with the exception of mass spectrometry analysis. Data were analyzed using one-way analysis of variance (ANOVA), and when significant differences were found, the multiple comparison Tukey-Kramer test was used (GraphPad InStat). Values of P<0.05 were considered statistically significant.

### List of accession numbers for mRNAs and proteins

mRNA eIF4G3: NM_001198801

Protein eIF4G3: NP_001185730

mRNA eIF4G1: NM_182917

Protein eIF4G1: NP_886553

mRNA Granzyme B: NM_004131

Protein GrB: NP_004122

mRNA Bid: NM_197966

Protein Bid: NP_932070

mRNA Granzyme A: NM_006144

Protein Granzyme A: NP_006135

mRNA Granzyme K: NM_002104

Protein Granzyme K: NP_002095

mRNA Caspase-3: NM_004346

Protein Caspase-3: NP_004337

## Results

### GrB cleaves the eukaryotic translation initiation factor 4 gamma 3

**Figure 1:**
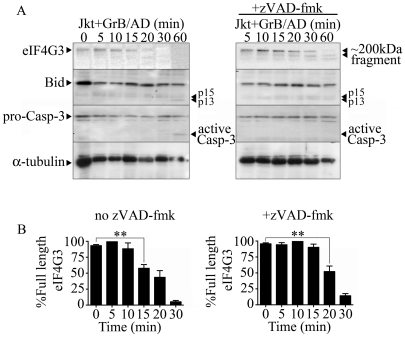
The eukaryotic translation initiation factor eIF4G3 is degraded by GrB (1 µg/ml) in Jurkat (Jkt) cells. (A) Representative Western blot analysis of detectable full-length eIF4G3 and Bid activation in Jurkat cells from time 0 (no GrB treatment) to 30 min post GrB/AD treatment (left panel) and in the presence of zVAD-fmk (right panel). (B) Densitometric analysis of eIF4G3 (A) where the eIF4G3/α-tubulin ratio was calculated and the highest densitometric value was 100%. Other values were plotted as a percentage of the maximum densitometric value. Statistical significance: p<0.01 (**); (*n* = 3 of 3 independent experiments).

To screen for novel GrB substrates, Jurkat cells were treated with GrB and then monitored for the resulting proteomic change. Proteomic analysis was conducted through 1D- and 2D-electrophoresis analysis (data not shown) and mass spectrometry. eIF4G3 was identified as a candidate using this approach as a spot that consistently disappear following GrB treatment. Through sequence analysis of eIF4G3, we found a sequence very similar to those cleaved by GrB in the known substrates Bid and caspase-3 (Table S1). Western blot analysis of eIF4G3 expression in Jurkat cells post GrB/AD treatment showed a significant reduction of detectable eIF4G3 by 15 min post treatment (Figure 1). This reduction of detectable eIF4G3 coincided with the appearance of a cleaved product. The observed degradation of eIF4G3 was not mediated by caspases since the caspase inhibitor zVAD-fmk did not prevent degradation of eIF4G3 (Figure 1A). EIF4G3 degradation was significant by 20 min post GrB/Ad treatment in the absence of caspase activity (Figure 1B left panel). To confirm that GrB activity was present, we monitored activation of Bid and Casp-3, two GrB substrates, during our time course analysis of eIF4G3 degradation. We found that Bid was activated at 60 min, as demonstrated by the presence of p15 and p13 fragments (Figure 1A). Active Casp-3 was also detected at 60 min post GrB/AD treatment (Figure 1A). Conversely, the lack of proteolytic cleavage of Casp-3 in the presence of zVAD-fmk indicated that zVAD-fmk inhibited the upstream caspases responsible for cleavage of Casp-3.

**Figure 2:**
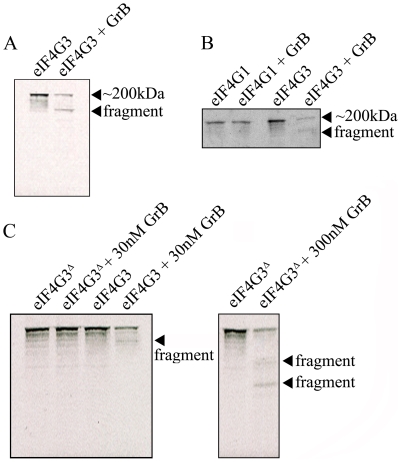
GrB-mediated cleave of eIF4G3 occurs at position D^1408^. (A) Autoradiography of eIF4G3 treated with 30 nM GrB for 30 min. Treatment of eIF4G3 (∼200 kDa) with 30 nM GrB generates a proteolytic fragment; (n = 3 of 3 independent experiments). (B) The eIF4G3 isoform, eIF4G1, is not degraded by GrB. Loaded protein levels for eIF4G1 and eIF4G3 lanes are identical to their corresponding GrB treatment lanes; (*n* = 3 of 3 independent experiments). To visualize eIF4G3 fragments, eIF4G3 lanes were loaded at a higher concentration. (C) Putative P1 residue D^1408^ of eIF4G3 was mutagenized to alanine to generate the mutant eIF4G3^Δ^. eIF4G3 and eIF4G3^Δ^ were synthesized *in vitro* and labeled with Met^S35^. These two proteins were treated with 30 and 300 nM GrB. Detectable degradation products are indicated with an arrow; (*n* = 3 of 3 independent experiments).

The location of the putative GrB cleavage site (P1 residue) of eIF4G3 is at aspartate 1408 (D^1408^); cleavage at this site would result in 2 fragments of approximately 180 kDa and 20 kDa in size. To test whether eIF4G3 is a substrate for GrB, the factor was synthesized using an *in vitro* transcription/translation system (IVTT) in the presence of radioactively labeled methionine (Met^S35^). The radioactively labeled eIF4G3 was then treated with GrB *in vitro* and analyzed by electrophoresis and subsequent autoradiography of the gels (Figure 2). Treatment of labeled eIF4G3 with 30 nM GrB resulted in the generation of a proteolytic fragment of eIF4G3, corresponding to the expected large fragment (Figure 2A). The small fragment expected from the cleavage of eIF4G3 was not detected in autoradiograms; this fragment was likely below the detectable limit of autoradiography as it contains only 3 radioactively labeled Met^S35^ residues per polypeptide, as opposed to 30 Met^S35^ residues present in the large proteolytic fragment. These results suggest that GrB cleaves eIF4G3 at D^1408^.

Factor eIF4G3 is similar to eIF4G1 and may share overlapping roles in translation. These two proteins share 46% sequence homology, but eIF4G1 lacks the D^1408^ residue. To test if eIF4G1 is also cleaved by GrB, eIF4G1 was treated with GrB under the same experimental conditions. Treatment of eIF4G1 with 30 nM GrB did not result in proteolytic cleavage of this isoform (Figure 2B).

D^1408^ is predicted to be the essential residue for cleavage by GrB. To confirm this, we mutagenized D^1408^ to alanine and tested the effect of this mutation on the proteolytic cleavage of eIF4G3 by GrB. This mutant (eIF4G3^Δ^) was resistant to 30 nM GrB treatment (Figure 2C). At a higher concentration of GrB (300 nM), eIF4G3^Δ^ was proteolytically cleaved by GrB. However, this reaction generated different degradation products suggesting cleavage at alternative aspartate residues (Figure 2C).

### GrB inhibits in vitro translation

**Figure 3:**
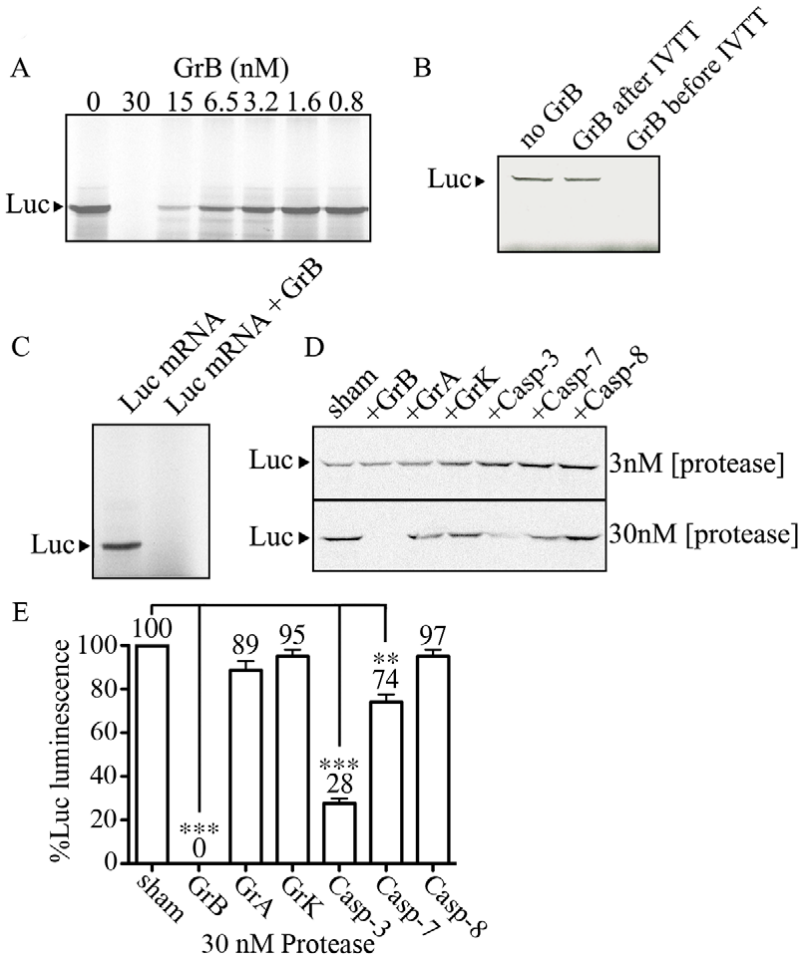
GrB inhibits *in vitro* translation of Luc. IVTT was used to synthesize Luc in the presence of Met^S35^. Labeled Luc protein was visualized by autoradiography. (A) A range of GrB concentrations (0–30 nM) were added to the IVTT master mix to test their effect on transcription/translation; (*n* = 3 of 3 independent experiments). (B) Detectable Luc was compared in the absence of GrB, and in the presence of 30 nM GrB added before or after IVTT; (*n* = 3 of 3 independent experiments). (C) Purified Luc mRNA (10 µg/ml) was used to synthesize Met^S35^-labeled Luc. 30 nM GrB prevents translation; (*n* = 3 of 3 independent experiments). (D) Two different concentrations of proteases (3 and 30 nM) GrB, granzyme A (GrA), granzyme K (GrK), Caspase-3 (Casp-3), Caspase-7 (Casp-7) and Caspase-8 (Casp-8) were tested in IVTT of Luc; (*n* = 3 of 3 independent experiments). (E) Luminescence generated by Luc in IVT reactions treated with 30 nM proteases, where sham-treated IVT reactions were considered 100%; Statistical significance: p<0.01 (**) and p<0.005 (***); (*n* = 3 of 3 independent experiments).

Our results showing that eIF4G3 is a substrate of GrB suggests that GrB can interfere with the translation machinery of target cells. We used the IVTT system to test whether GrB can inhibit protein synthesis. The IVTT system uses a master mix that contains transcription components from bacteriophage and translational components from rabbit reticulocyte lysate. We used a luciferase (Luc) DNA template for transcription and subsequent translation in the presence of Met^S35^. Luc protein synthesis was monitored by SDS-PAGE followed by autoradiography. Next, the *in vitro* synthesis of Luc was conducted in the presence or absence of GrB. GrB was added to the IVTT master mix prior to adding Luc DNA. GrB treatment reduced synthesis of Luc protein in a concentration-dependent manner (Figure 3A). At a concentration of 30 nM of GrB, Luc protein synthesis was no longer detectable with this technique. Adding GrB to the reaction following *in vitro* synthesis of Luc did not degrade Luc protein (Figure 3B), indicating that the changes observed in Figure 3A were a result of changes in expression of Luc and not GrB-dependent degradation of Luc.

In the IVTT system, GrB could be targeting proteins involved in transcription, translation, or both. In order to determine if GrB affects mammalian translation independent of transcription, we used purified Luc mRNA and performed *in vitro* synthesis of Luc protein from mRNA. This approach converted the IVTT system into an *in vitro* translation (IVT) system. To ensure that the *in vitro* synthesis of Luc protein was restricted to translation, DNase was included in all IVT reactions to eliminate any possible DNA contaminants. Subsequently, when we added RNase, the synthesis of Luc protein was completely prevented, demonstrating that detectable Luc is a result of mRNA translation under our IVT system (Figure S1). In the presence of 30 nM GrB, IVT was prevented (Figure 3C). These results demonstrated that GrB was blocking *in vitro* synthesis of Luc by interfering at the translational level. To test whether other proteases had the same effects on translation, we tested GrB, GrA, GrK, Casp-3, Casp-7 and Casp-8 at concentrations of 3 and 30 nM in the same system (Figure 3D). GrB was the only enzyme able to completely prevent IVT of Luc under these conditions. Luminescence from these reactions indicated that 30 nM GrB, Casp-3 and Casp-7 resulted in a significant decrease of Luc activity, whereas GrA, GrK and Casp-8 showed no significant alteration of Luc activity (Figure 3E).

### GrB prevents in vitro translation by targeting eIF4G3

**Figure 4:**
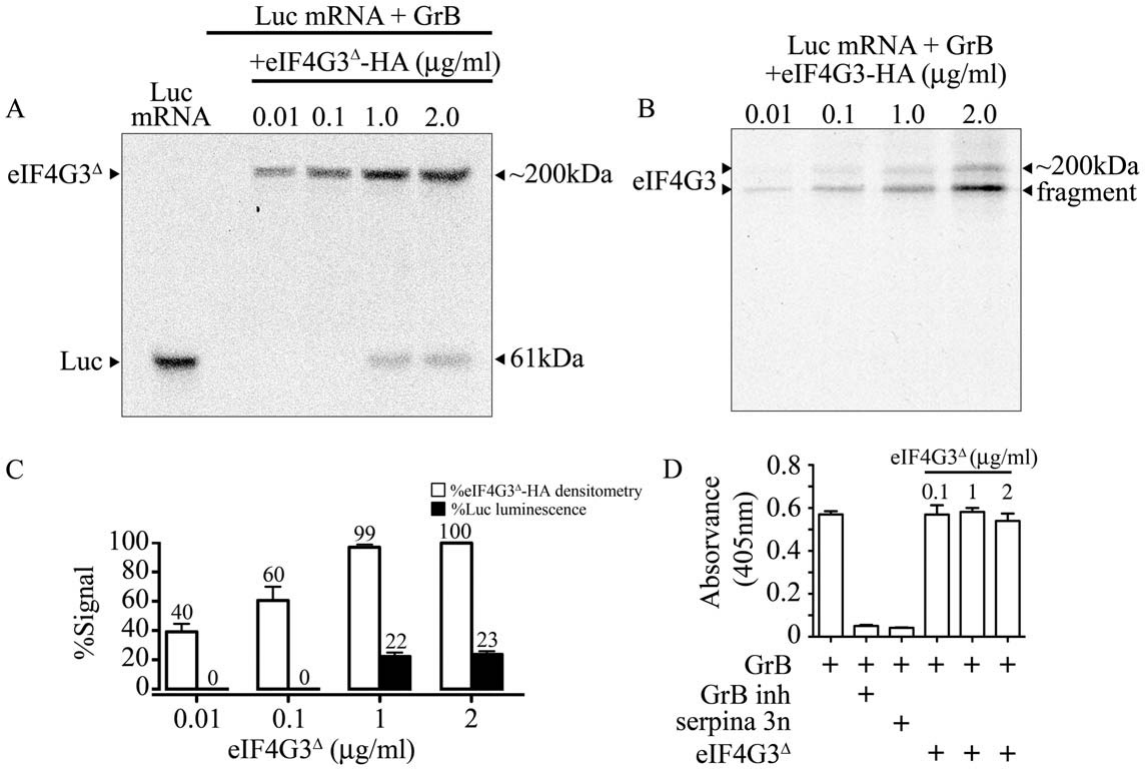
GrB resistant eIF4G3^Δ^ can restore *in vitro* translation of Luc. (A) Translation of Luc mRNA was monitored by autoradiography. In the presence of 30 nM GrB, translation of Luc is blocked. Purified eIF4G3^Δ^ -HA (0.01–2 µg/ml) was added the reaction after GrB treatment to recover Luc *in vitro* synthesis. Luc shows as a band at 61 kDa and radio-labeled eIF4G3^Δ^ -HA shows as a band at ∼200 kDa; (*n* = 3 of 3 independent experiments). (B) Same experiments as in (A) but with purified wild-type eIF4G3-HA; (*n* = 3 of 3 independent experiments). (C) Luminescence generated by Luc IVT in (A) was measured and expressed as %Luc luminescence, where expression of Luc with no treatment was considered 100%. Densitometric analysis was conducted for eIF4G3^Δ^ -HA, and expressed as %eIF4G3^Δ^ -HA densitometry, where densitometric values for Luc with no treatment was considered 100%; (*n* = 3 of 3 independent experiments). (D) GrB activity colorimetric assay. GrB inh and purified serpina 3n were used as positive controls to inhibit GrB activity. Three different concentrations of eIF4G3^Δ^ (0.1, 1 and 2 µg/ml) did not inhibit GrB activity; (n = 3 of 3 independent experiments).

To determine whether GrB cleavage of eIF4G3 is responsible for the GrB-mediated translational inhibition of Luc, we tested whether supplementing the IVT reaction with the GrB-resistant mutant eIF4G3^Δ^ would regain translation of Luc. Mutant eIF4G3^Δ^ and wild-type eIF4G3 were HA-tagged and purified using affinity chromatography (Fig S2). Purified GrB-sensitive eIF4G3 and GrB-resistant eIF4G3^Δ^ were then added to the IVT of Luc, in the presence of GrB. Analysis of Luc protein synthesis and luminescence showed that recovery of translation was observed with eIF4G3^Δ^ (Figure 4A), but not the wild-type protein (Figure 4B). Adding 1 and 2 µg/ml of eIF4G3^Δ^-HA to the GrB-treated IVT led to 22% and 23% recovery of Luc luminescence, respectively, when compared to the luminescence in untreated Luc IVT (Figure 4C). The ability of GrB-resistant eIF4G3^Δ^ to rescue GrB-damaged translation machinery indicates that cleavage of eIF4G3 by GrB is a key event in translational inhibition. Other substrates may be involved but eIF4G3 is important. To exclude the possibility that eIF4G3^Δ^ rescues translation by inhibiting the activity of GrB, we performed a GrB enzymatic assay in the presence of 0.1 – 2 µg/ml of eIF4G3^Δ^. Our positive controls for the assay included GrB inh and serpina 3n, both of which significantly reduced GrB activity (Figure 4D). However, eIF4G3^Δ^ did not affect GrB activity at all concentrations tested (Figure 4D).

### GrB-dependent translational arrest is caspase-independent

**Figure 5:**
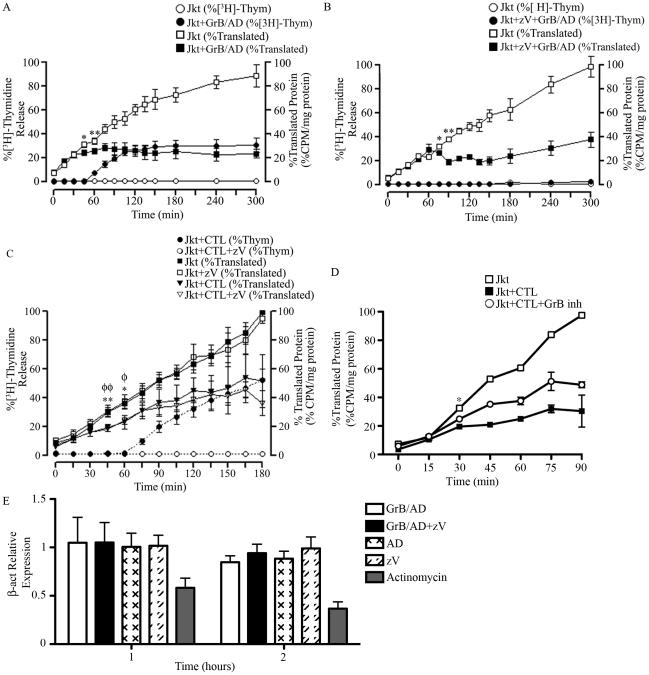
GrB or CTL treatment decrease translational rate in Jurkat cells independently of caspase-mediated DNA fragmentation. (A and B) Jurkat cells were treated with GrB (1 µg/ml) for 300 min. At 15 min interval, cells were assessed for DNA fragmentation (%[^3^H]-Thymidine Release) and translational rate (% Translated Protein). (B) The pan-caspase inhibitor zVAD-fmk (zV) was used to evaluate the role of caspases. (For (A) and (B); *n* = 4 of 4 independent experiments). (C) Jurkat cells were treated with CTL for 180 min. As in (A) and (B), DNA fragmentation and translational rate was assessed at 15 min intervals. Statistical significance: p<0.05 (* and φ) p<0.01 (** and φφ); *n* = 3, where * compares statistical significance of % translated protein of Jurkat cells+zVAD-fmk (Jkt+zV) vs. Jurkat cells+zV+CTL; and φ compares Jkt vs. Jkt+CTL; (n = 3 of 3 independent experiments). (D) Jurkat cells were treated with CTL for 90 min in the presence of the GrB inhibitor L-038587-00Y001 (GrB inh). %Translated protein was assessed at 15 min intervals. Statistical significance: p<0.05 (* and φ), where * compares statistical significance of % translated protein of Jurkat cells+CTL vs. Jurkat cells+CTL+L−038587−00Y001; (n = 3 of 3 independent experiments). (E) qPCR analysis of β-actin (β-act) relative expression at 1 and 2 hr post GrB/AD treatment; (n = 3 of 3 independent experiments).

It is well established that GrB-induced DNA fragmentation, a hallmark of apoptosis, in Jurkat cells begins at around 30–60 min and reaches a plateau at around 120 min [38]. We confirmed this observation by measuring %[^3^H]-thymidine release in Jurkat cells in response to GrB (Figure 5A). Next, a time course analysis of GrB-induced translational inhibition in relation to the timing of DNA fragmentation was performed. The incorporation of Met^S35^ into newly synthesized proteins at 15 min intervals for 300 min was measured. A significant reduction in Met^S35^-labeled proteins was detected at 45 min post-GrB treatment, whereas significant DNA fragmentation was detected at 60 min post-GrB treatment. The lowered translational rate did not recover after 45 min (Figure 5A). These results show that translational arrest precedes the onset of apoptosis as measured by DNA fragmentation, which was consistent in all 4 independent experiments.

GrB activates several caspases including caspase-3, 7, 8, 9 and 10. To determine if the observed reduction in the translational rate was mediated by the activation of caspases, we used the pan-caspase inhibitor zVAD-fmk. As expected, treatment of Jurkat cells with zVAD-fmk prevented GrB-mediated DNA fragmentation for the entire 300 min (Figure 5B). The addition of zVAD-fmk delayed the onset of translational inhibition by GrB to 75 min (Figure 5B) in comparison to 45 min (Figure 5A) in the absence of zVAD-fmk. However, translational inhibition by GrB was maintained even in the presence of zVAD-fmk as evidenced by the plateau in translation after 75 min (Figure 5B). These findings suggest that GrB-induced arrest in translation can occur independently of caspase activity, but that there may be cooperation between caspase-dependent and caspase-independent pathways. These results agree with our findings that GrB-mediated degradation of eIF4G3 is delayed in the presence of zVAD-fmk (Figure 1A).

### Human CTLs reduce translational rate in Jurkat cells

To confirm our findings using a more physiological system, we tested translational changes using cell-mediated killing in an allo-response [39]. CTL granules contain a complex proteome that includes an abundance of GrB. Jurkat cells were treated for 180 min with a human CTL line and assessed for Met^S35^ incorporation at 15 min intervals. As expected, CTL-induced apoptosis in Jurkat cells was prevented in the presence of zVAD-fmk (Figure 5C). This effect was also observed in Annexin V binding experiments (Figure S4B). Independent of zVAD-fmk treatment, CTLs induced a significant reduction of translation by 45 min in Jurkat cells (Figure 5C). These results indicated that CTL-mediated reduction of translation in target cells takes place independently of caspases. In the presence of the specific GrB inhibitor (L-038587-00Y001), inhibition of CTL-mediated translational was significantly decreased by 30 min post CTL treatment (Figure 5D). To confirm that GrB does not affect transcription, we performed qPCR of β-actin in Jurkat cells in the presence and absence of GrB/AD. Addition of GrB had no significant effect on mRNA transcription of β-actin in Jurkat cells; the well-known transcriptional inhibitor actinomycin D significantly reduced transcription in this system (Figure 5D).

To measure the effect of CTLs on Jurkat eIF4G3 levels, eIF4G3 protein levels were measured by Western blot. When CTLs were added to Jurkat cells, a time-dependent reduction in eIF4G3 levels was observed (Figure S3A). After 10 min treatment, the eIF4G3 signal was not significantly different from CTL alone (Figure S3B). This suggests that CTL treatment results in degradation of eIF4G3 in Jurkat cells. Furthermore, pre-treating Jurkat cells with zVAD-fmk did not have any significant effect on detected levels of eIF4G3, indicating that eIF4G3 degradation is caspase-independent (Figure S3B).

### Arrest of VV protein synthesis by GrB in Jurkat cells correlates with GrB-dependent degradation of eIF4G3

**Figure 6:**
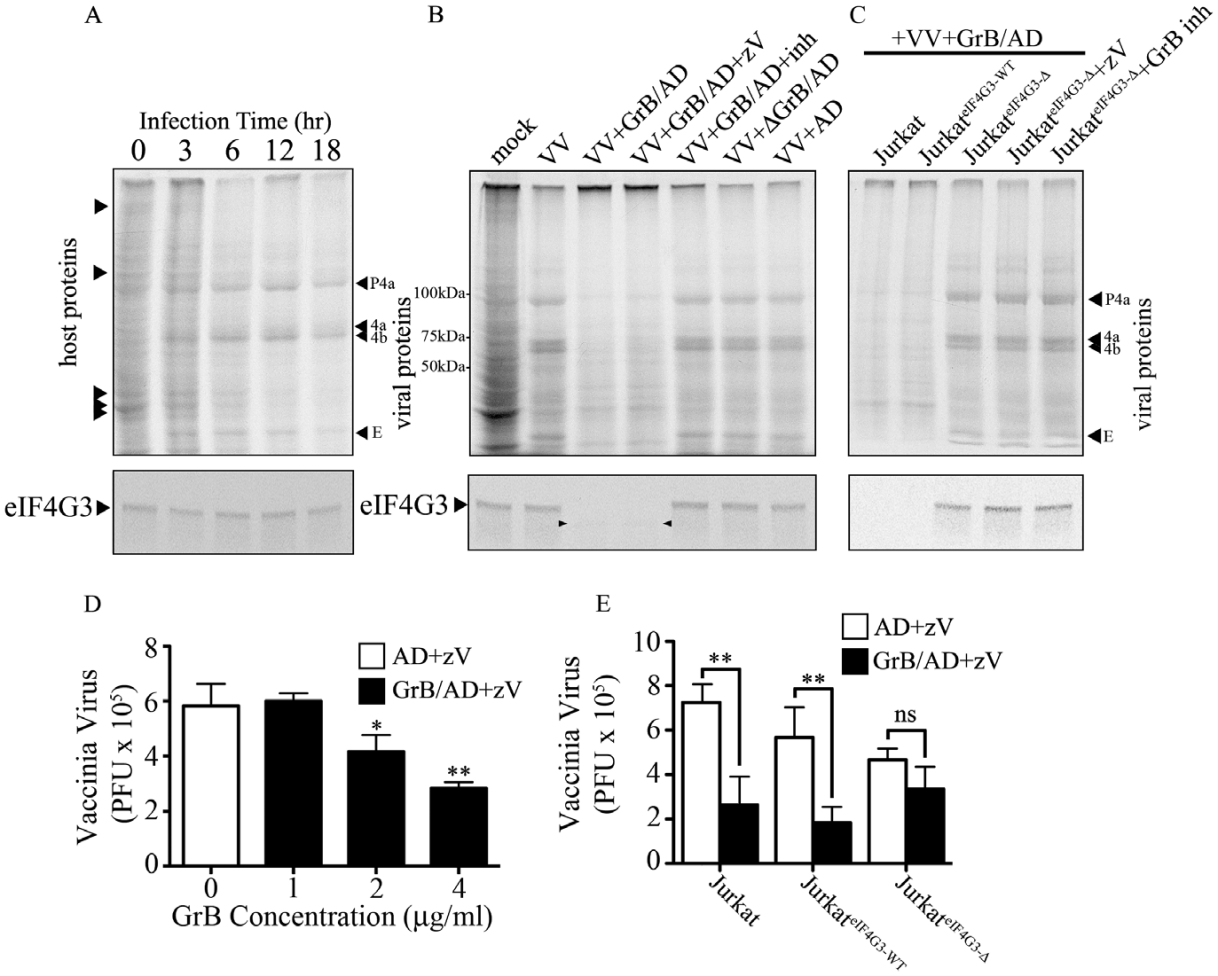
Inhibition of VV production by GrB is mediated by cleavage of eIF4G3. (A) Jurkat cells were infected with VV for 0 to 18 hr. Metabolic labeling was assessed by Met^S35^ incorporation and viral protein synthesis was monitored by autoradiography analysis. Arrowheads on the left hand side of the radiogram indicate host proteins and arrowheads on the right hand side of the radiogram indicate VV proteins. Late VV proteins P4a, 4a, 4b and early VV protein E are shown. Western blot analysis of eIF4G3 from each time point is shown below the radiogram. (B) Jurkat cells were infected for 12 hr with no virus (mock) or with VV. Infected cells were co-treated with AD, GrB/AD, GrB/AD+zVAD−fmk (zV), GrB/AD+GrB inhibitor (inh) and heat inactivated GrB+untreated AD (ΔGrB/AD). Western blot analysis of eIF4G3 from each experimental condition is shown below the radiogram. Arrowheads in the Western blot image point to eIF4G3 degradation product; (*n* = 4 of 4 independent experiments). (C) Jurkat cells, Jurkat cells transformed with wild-type eIF4G3 (Jurkat^eIF4G3-WT^) and Jurkat cells transformed with GrB-resistant mutant (Jurkat^eIF4G3-Δ^) were infected with VV for 12 hr (D) The effect of GrB concentration (1–4 µg/ml) on VV replication was tested. AD and zV concentrations were the same in all treatments; Statistical significance: p<0.05 (*) and p<0.01 (**); where * compares 2 and 4 µg/ml GrB vs. 0 µg/ml GrB; (*n* = 3 of 3 independent experiments). (E) VV replication in untreated Jurkat cells, Jurkat^eIF4G3-WT^, and Jurkat^eIF4G3Δ^, was measured by plaque assay. Statistical significance: p<0.01 (**); (*n* = 3 of 3 independent experiments).

Thus far, the experiments looked at global translational inhibition by GrB. However, the major point of our model is that viral protein synthesis will be blocked early to halt the generation of infectious virus. To test this hypothesis, we infected Jurkat cells with VV and tested viral protein synthesis in the presence of GrB. During an infection, VV will hijack the host translational machinery for viral production. Thus, a successful VV infection is confirmed when only VV proteins are detected. We monitored protein expression by metabolic labeling. By 12 hr of infection, there was a near absence of host proteins and detection of late VV proteins - precursor 4a (P4a), processed structural proteins 4a and 4b and the early protein E (Figure 6A upper panel). To test whether late VV protein expression is affected by GrB treatment, we infected Jurkat cells for 12 hr, treated with GrB for 1 hr, and then performed metabolic labeling analysis. Mock infected cells and VV infected cells were run to compare host versus VV protein expression (Figure 6B upper panel). GrB treatment reduced VV protein expression, which was not affected by the caspase inhibitor zVAD-fmk (Figure 6B). Inactivation of GrB with a specific inhibitor (L-038587-00Y001) or heat inactivation prior to treatment reversed VV protein expression, while AD treatment alone did not affect VV protein expression (Figure 6B). To test whether VV protein expression was a direct result of GrB-dependent degradation of eIF4G3, we transfected Jurkat cells and generated stable clones of wild-type eIF4G3 (Jurkat^eIF4G3-WT^) and mutagenized GrB-resistant eIF4G3^Δ^ (Jurkat^eIF4G3Δ^). GrB/AD treatment following 12 hr of VV infection prevented the formation of detectable VV proteins in Jurkat cells and in the Jurkat^eIF4G3-WT^ clone. However, expression of the GrB-resistant eIF4G3^Δ^ reversed the GrB-mediated protection, resulting in formation of late VV proteins (Figure 6C). These results provide strong mechanistic evidence supporting a model where GrB-mediated degradation of eIF4G3 prevents VV protein expression.

In the absence of GrB, eIF4G3 protein expression did not change significantly from 0 to 18 hr post infection (Figure 6A lower panel), while GrB-dependent downregulation of viral protein expression correlated with a decrease in eIF4G3 levels (Figure 6B–C lower panel). This confirmed that cleavage of eIF4G3 coincides with VV protein expression.

### GrB blocks formation of infectious VV particles by proteolytic cleavage of eIF4G3

To study the effect of GrB on the formation of VV particles, we used a plaque assay to measure VV titres from infected Jurkat cells. In our optimization time course experiments, we found that significant VV production was detected starting at the 6 hr time point (data not shown). Thus, Jurkat cells were infected with VV for 6 hr, then treated for 1 hr with increasing concentrations of GrB in the presence of AD (10 PFU/cell) and zVAD-fmk. Following treatment, cells were lysed and the cytosolic fraction was used to infect BGMK cells in a plaque assay. GrB significantly reduced the production of VV particles from Jurkat cells at 2 and 4 µg/ml (Figure 6D). The metabolic labeling results shown in Figure 6A–C only detected VV proteins synthesized after GrB treatment and subsequent addition of [^35^S]methionine and [^35^S]cysteine. Thus, only newly synthesized (from metabolic labeling and onwards) VV proteins, not total VV proteins, are detected in Figure 6A–C. The plaque assay used in Figure 6D–E is more sensitive and relevant to our model than the metabolic labeling of VV proteins. However, this assay cannot distinguish between VV proteins present before and after GrB treatment. This is evident in our time course analysis of VV protein synthesis, where we detect VV proteins at 6 hr post-infection (Figure 6A). By the time GrB is added at 6 hr post-infection as shown in Figure 6D–E, these VV proteins have already been made. Although GrB treatment quickly halts VV protein synthesis, the already existing VV proteins are not degraded by the addition of GrB and are still capable of generating new VV particles. This fundamental difference between the two assays led us to identify that 2–4 µg/ml of GrB was needed to induce the expected GrB-mediated reduction in VV titers (Figure 6D).

Expression of GrB-resistant eIF4G3^Δ^ resulted in significant recovery of GrB-dependent translational halt of host protein synthesis by 2 hr (Figure S4C). Similar results were obtained in CTL-dependent translational halt (data not shown). The critical experiment in this study was to test the production of VV from Jurkat^eIF4G3Δ^ cells. We predicted that GrB would be unable to degrade eIF4G3^Δ^ in these cells, thus allowing VV replication in the presence of GrB. Expression of GrB-resistant eIF4G3^Δ^ reversed the effects of GrB on VV infection, as the production of VV particles in Jurkat^eIF4G3Δ^ was not affected by GrB treatment (Figure 6E). These experiments indicate that eIF4G3 is a critical GrB substrate that is targeted to halt production of infectious VV.

## Discussion

When a cell is infected with a virus, it becomes a factory for production of new infectious virus. Immune destruction of the infected cell ultimately puts a halt to this, but during the killing process, large amounts of virus could still be produced. It seems logical to propose that a successful strategy to eradicate virus infection should also bring a dramatic halt to virus production. The function of GrB to proteolytically activate pro-apoptotic factors like caspase-3 and Bid has been extensively investigated [40]. In this study, we describe a novel role of GrB to block protein synthesis in a VV-infected cell independently of GrB's role in triggering apoptosis. This strategy transforms the host into a deficient system for viral replication, thus preventing viral spread. Importantly, we definitively showed that eIF4G3 is the critical substrate of GrB affected in protein translation.

Proteomic approaches led us to eIF4G3 as a putative GrB substrate. Within the C-terminal domain of eIF4G3, there is a sequence similar to that present in Bid and caspase-3, where GrB cleaves these two substrates (Table S1). The only differing amino acid in eIF4G3 is the P2 residue (S^1407^); however, this serine is found at the same P2 location in other reported GrB substrates like centromeric protein b (CENP-B) (Table S1). eIF4G3 was cleaved *in vitro* by GrB and in Jurkat cells treated with either GrB or human CTLs. When we mutagenized the critical aspartate residue in eIF4G3 to alanine, the resulting mutant (eIF4G3^Δ^) was GrB-resistant. This evidence demonstrated that D^1408^ is the location at which GrB cleaves eIF4G3.

In order to test whether GrB inhibited protein synthesis, we used an IVTT system programed by a luciferase gene. When the IVTT master mix was pretreated with 30 nM GrB, we were unable to detect protein synthesis or Luc activity. Since this could occur as a result of disruption of transcription or translation, the IVTT system was modified to specifically look at translation. This was achieved by first performing a regular IVTT of Luc. Then, Luc-mRNA was purified and used as a starting material in a new synthesis reaction. An important experimental parameter was to include DNase in the subsequent IVT reactions, to prevent transcription from any possible DNA that could be present in the master mix as provided by the manufacturer. This approach was used to determine that GrB was targeting and disrupting translation.

Following treatment with GrB, the disabled protein synthesis system was restored by supplementing the system with eIF4G3^Δ^, but not with eIF4G3, demonstrating that cleavage of eIF4G3 by GrB is a critical event in translation inhibition. Recovery of translation was only partial, thus it is possible that GrB treatment directly or indirectly targets additional proteins involved in translation. The capacity of GrB to inhibit *in vitro* protein synthesis of Luc was confirmed in Jurkat cells. Within 45 min post-GrB treatment, translation was completely stopped with no significant recovery for up to 12 hr (Figure 5 and Figure S4C).

When we treated VV-infected Jurkat cells with GrB, there was a significant reduction in the synthesis of viral proteins and new infectious VV particles. Stable transfection of Jurkat cells with the GrB-resistant eIF4G3^Δ^ generated clones that were significantly more susceptible to VV infection in the presence of GrB. This experimental approach demonstrated that GrB blocks cap-dependent translation by specifically targeting eIF4G3 and cleaving this protein significantly reduces VV production.

In eukaryotes, translational regulation is a critical method to control gene expression. With the exception of some genes, the majority of eukaryotic mRNAs are translated through cap-dependent translation. This process can be separated into the stages of initiation, elongation, termination and ribosomal recycling. Translational initiation is the stage where the majority of regulation takes place. Global regulation of translation initiation involves eIF inactivation [21]. This approach is used by cells during starvation or periods of stress and is employed during apoptosis to allow expression of key regulators. Although translation inhibition has been reported during apoptosis, we have shown for the first time that GrB alone can initiate translational shutoff and in doing so, prevent VV replication.

In this study, we used Jurkat cells. These are commonly used as targets in CTL and granzyme assays since they die fairly quickly as revealed in a 4 hr chromium assay. This is in contrast to other target cells, particularly primary cells in which the time to induce cell death by GrB is much longer. We have estimated that one VV particle infecting a single cell can generate one hundred new viral particles in 24 hr. Thus, over this time frame, the inhibition of virus production will have significant effects. In addition, many viruses produce proteins that can inhibit apoptosis, thereby prolonging the generation of infectious virus. Although not the focus of this work, the inhibition of protein synthesis may also have implications for the control of apoptosis during viral infection.

The roles of all three eIF4G isoforms are unclear. It is accepted that isoform eIF4G2 functions to inhibit translation, while isoforms eIF4G1 and eIF4G3 are involved in initiation of translation. Although eIF4G1 and eIF4G3 contain similar domains, they only share 46% of their sequence. Interestingly, when cap-dependent translation is inhibited by viruses, degradation of eIF4G3 is the rate limiting step and not eIF4G1 [23], highlighting the importance of eIF4G3 in the regulation of translation. Using a positional proteomic approach, it was reported recently that GrB can cleave eIF4G1 at an unusual recognition sequence - CGPD^665^F [41]. While it is possible that GrB can cleave both eIF4G1 and eIF4G3, we found that GrB was unable to cleave eIF4G1 in our IVTT proteolytic assays.

Cleavage of eIF4G3 at D^1408^ results in a protein with a truncated C-terminal domain. We postulate that an undescribed function of this domain exists. The isoform eIF4G2 acts as general repressor of translation by inhibiting the functions of eIF4G1 and eIF4G3. Over-expression of eIF4G2 can inhibit both cap-dependent and IRES-mediated protein synthesis [42], [43]. It is possible that the truncated portion of eIF4G3 inhibits the function of eIF4G1 similar to eIF4G2, thus having a dominant negative effect on translation. In concordance with previous studies, we propose that the large proteolytic fragment of eIF4G3 might have translational inhibitory properties distinct from degradation of eIF4G3. Alternatively, cleavage may result in a conformational change of eIF4G3 folding that is responsible for the loss of its function. Our finding that GrB inactivates eIF4G3 through proteolytic cleavage with resulting decrease in protein translation agrees with the current model of eIF4G3 function. During infection by VV, GrB could also inhibit translation of specific viral proteins that limit host responses. For example, by blocking synthesis of CrmA, a serpin from VV that inhibits caspase activation, GrB would allow caspases to perform their apoptotic functions.

Synthesis of eIF4G3 and eIF4G1 can occur through the use of internal IRES elements, independent of cap-mediated translation [44], [45]. In concordance with these results, we found that eIF4G3 downregulation was not detected during infection with VV, although total host protein synthesis was significantly reduced (Figure 6A). These results also agree with existing evidence that VV preserves the eIF4F complex [24] and that VV is unable to replicate in the absence of eIF4G3 (a subunit of the eIF4F complex) [23], [25].

A number of studies have reported caspase-3-dependent degradation of eIF4G1 in cells undergoing apoptosis [46], [47], [48]. In the current study, GrB did not degrade eIF4G1 *in vitro*; however, GrB could degrade both eIF4G3 and eIF4G1 in cells through direct degradation of eIF4G3 and indirectly through activation of caspase-3. While it has been shown that caspase-3 cleaves eIF4G3 [49], we found that inhibition of caspases by zVAD-fmk did not prevent GrB-dependent degradation of eIF4G3 or arrest of translation. However, zVAD-fmk changed the kinetics of eIF4G3 degradation (Figure 1A–B) and host translational arrest induced by GrB from 45 min with GrB alone to 75 min in the presence of zVAD-fmk. This suggests that there is cooperation between caspase-dependent and caspase-independent pathways mediated by GrB in the arrest of protein synthesis. We propose a model where GrB inhibits translation by 1) proteolytic cleavage of eIF4G3 and 2) indirect degradation of eIF4G1 through activation of caspase-3.

Since the discovery of GrB, a significant amount of work has been carried out to decipher the role of GrB in CTL-mediated killing. We now recognize that GrB, directly and through the mitochondrial pathway, activates key substrates like caspase-3 and Bid to induce apoptosis [40]. In this study, we present findings that suggest a dramatic change of paradigm with regards to the roles of GrB. The ability of GrB to halt the host translational machinery is a novel strategy to prevent viral replication in a host cell targeted by CTLs. This mechanism of GrB has not been described for other granzymes within the granzyme family of proteases; specifically, GrA and GrK were unable to inhibit translation. Although GrH has been shown in literature to inhibit viral translation by targeting the RNA-binding protein La, they did not demonstrate an effect of GrH on host translational machinery [19]. Whether the effect of GrB on translation is due to proteolytic inactivation of eIF4G3 or that cleavage of eIF4G3 results in products with new suppressive functions on translation remains to be determined. Our findings unveil a new antiviral axis of GrB function to fight viral infection by targeting translation.

## Supporting Information

Figure S1 *In vitro* translation of Luc is performed from purified Luc mRNA. 1 µg/ml of Luc DNA (vector) was used to synthesize Luc protein *in vitro,* showing transcription and translation of Luc. In the presence of DNase, Luc protein synthesis was blocked. Purified Luc mRNA is used to synthesize Luc protein from mRNA, thus restricting the process to translation. In the presence of DNase, to prevent any Luc DNA contamination. Higher concentrations of mRNA resulted in higher amount of translated Luc protein. This reaction was abrogated in the presence of RNase. This approach converted the IVTT system into an *in vitro* translation (IVT) system.
(TIF)

Figure S2 Purification of HA-tagged eIF4G3. (A) Diagram representing reported domains within the eIF4G3 sequence and the location of the HA-tag. GrB cleavage site is indicated with an arrow (D^1408^). (B) To monitor the effectiveness of the chromatographic approach, we ran SDS-PAGE followed by silver staining of the samples at different stages of the purification. Naïve MM indicates a master mix that was not altered in any way. MM eIF4G3^Δ^-HA indicates master mix after IVTT of eIF4G3^Δ^-HA. 1^st^, 2^nd^ and 3^rd^ flow through refer to 3 consecutive washes of the columns. Purified eIF4G3^Δ^-HA refers to eluted fraction (C) The same gel was then dried and analyzed by autoradiography. (D) The silver stained gel and the autoradiography film were superimposed and shown at higher resolution. The same approach was used for HA-tagged wild-type eIF4G3. (E) Purified products were detected by Western blot analysis at a concentration of 0.1 µg of purified protein per lane.
(TIF)

Figure S3 CTL-treatment of Jurkat cells induces eIF4G3 degradation. (A) Expression of eIF4G3 was accessed by Western blot analysis in the first 30 min after treating Jurkat cells with CTL in the absence of zVAD-fmk (no zV) or in its presence (+zV). (B) Densitometric analysis of (A) where the eIF4G3/β-actin (β-act) ratio was calculated and the highest densitometric value was a 100%. The data was plotted as a percentage of the maximum densitometric value. Statistical significance: p<0.01 (**); (*n* = 6 of 6 independent experiments).
(TIF)

Figure S4 GrB and CTL-induced changes in apoptosis and translation. Jurkat cells were treated with (A) GrB/AD and (B) CTL for 24 hr. %Annexin positive cells were measured at 1 hr intervals up to 6 hr and then at 12 hr and 24 hr. Sham treatment included AD at a concentration of 10 PFU/cell. For (A and B) statistical significance of p<0.01 (**) is shown where * compares statistical significance of Jurkat cells treated with CTL vs. Jurkat cells treated with CTL in the presence of zV; (n = 5 of 5 independent experiments). (C–E) Untreated Jurkat cells, Jurkat cells transfected with wild-type eIF4G3 (Jurkat^eIF4G3-WT^) and Jurkat cells transfected with the GrB-resistant eIF4G3 mutant (Jurkat^eIF4G3Δ^) were treated with GrB and AD (GrB/AD). (C and D) DNA fragmentation was measured as %[3H]-Thymidine release (C) in the absence of zVAD-fmk (no zV) and (D and E) in the presence of zVAD-fmk (+zV). (E) %Translated protein was measured; Statistical significance: p<0.05 (* and φ) p<0.01 (** and φφ); where * compares statistical significance of Jurkat^eIF4G3-WT^+GrB/AD vs. Jurkat^eIF4G3Δ^+GrB/AD; and φ compares statistical significance of Jurkat+GrB/AD vs. Jurkat^eIF4G3Δ^+GrB/AD; (For C–E, n = 3 of 3 independent experiments).
(TIF)

Table S1 Sequence alignment for GrB substrate cleavage sites, related to Figure 2. GrB cleaves these substrates between the Aspartate at the P1 and the P1' residue. The 5 residues shown for each substrate (P4 to P1') are critical for GrB catalytic cleavage. The GrB substrates are Caspase-3 (casp-3), BH3 Interacting domain Death agonist (Bid), eukaryotic initiation factor 4 gamma 3 (eIF4G3), and centromeric protein b (CENP-B).
(DOC)
