# Supplementary figures and images for: Granzyme B Inhibits Vaccinia Virus Production through Proteolytic Cleavage of Eukaryotic Initiation Factor 4 Gamma 3
Source: PLoS Pathog. 2011 Dec 15;7(12):e1002447. doi: 10.1371/journal.ppat.1002447 (PMC3240606; doi:10.1371/journal.ppat.1002447)

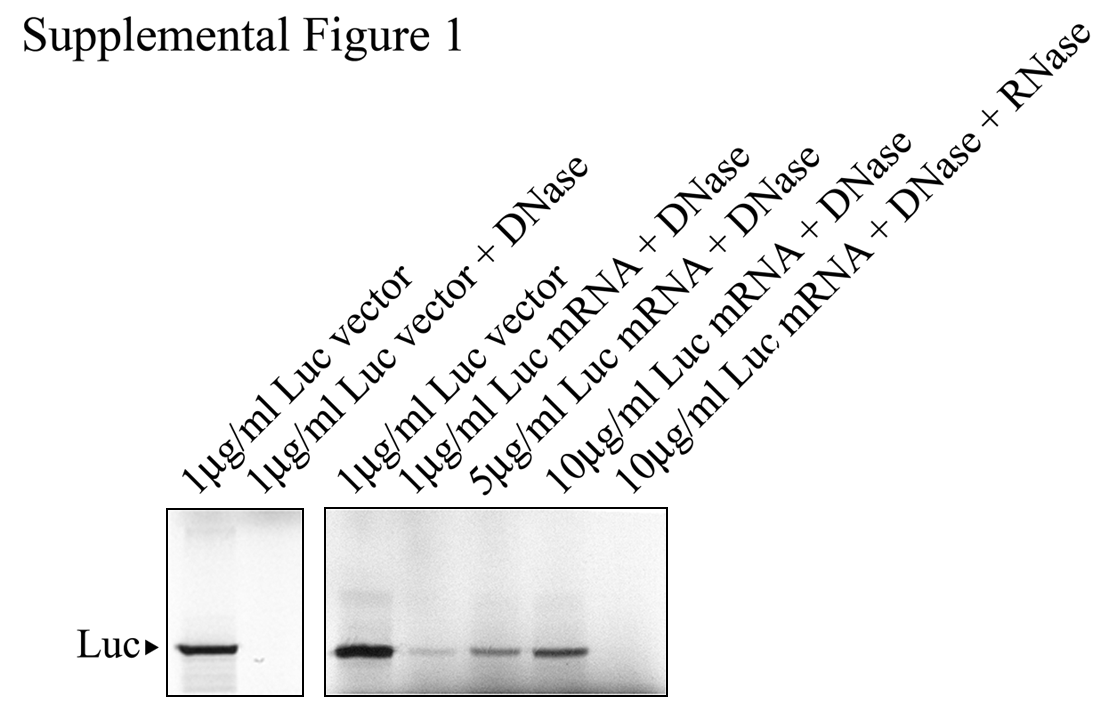

Supplement: Figure S1 — In vitro translation of Luc is performed from purified Luc mRNA. 1 µg/ml of Luc DNA (vector) was used to synthesize Luc protein in vitro, showing transcription and translation of Luc. In the presence of DNase, Luc protein synthesis was blocked. Purified Luc mRNA is used to synthesize Luc protein from mRNA, thus restricting the process to translation. In the presence of DNase, to prevent any Luc DNA contamination. Higher concentrations of mRNA resulted in higher amount of translated Luc protein. This reaction was abrogated in the presence of RNase. This approach converted the IVTT system into an in vitro translation (IVT) system. (TIF) [file ppat.1002447.s001.tif]

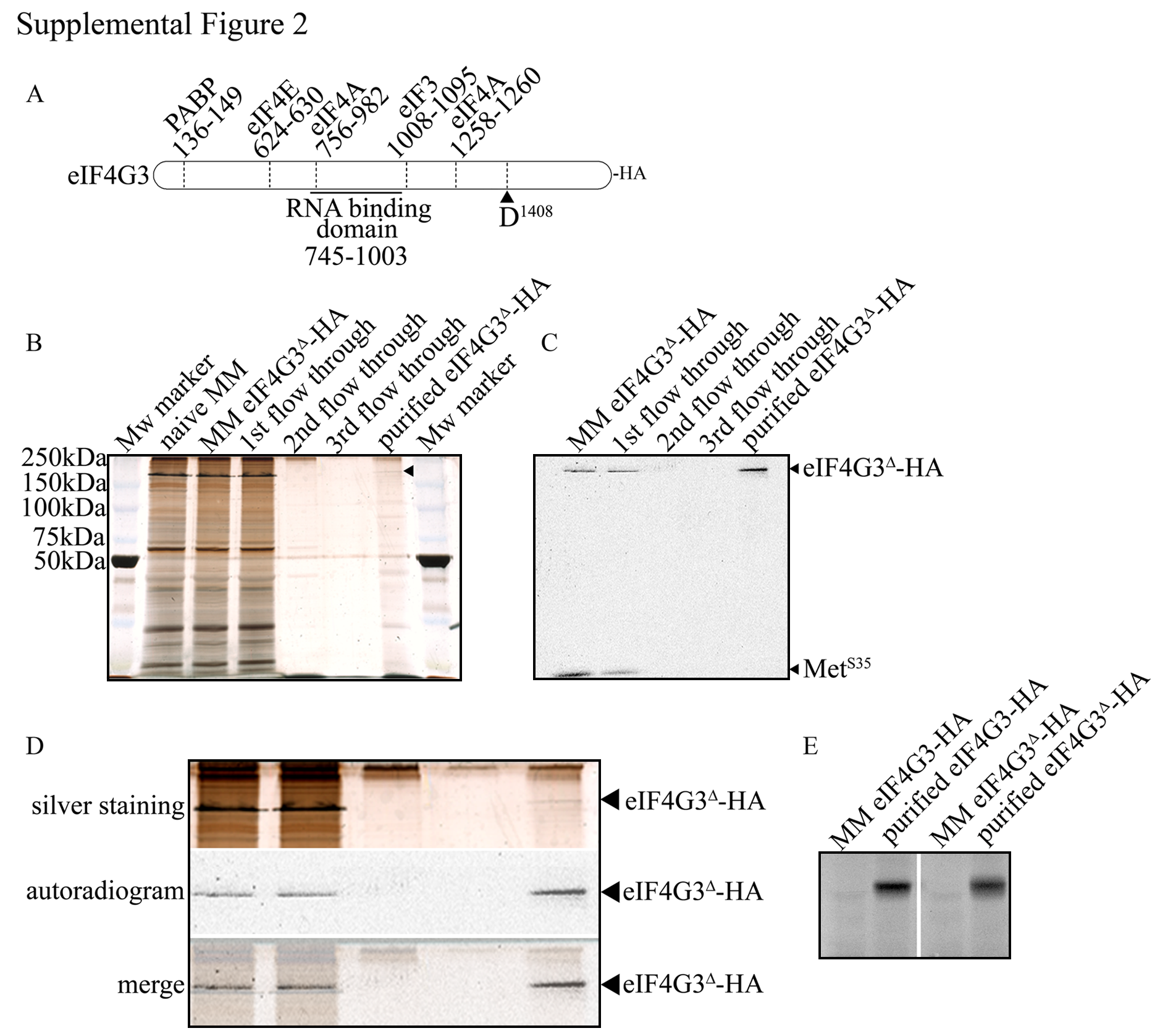

Supplement: Figure S2 — Purification of HA-tagged eIF4G3. (A) Diagram representing reported domains within the eIF4G3 sequence and the location of the HA-tag. GrB cleavage site is indicated with an arrow (D1408). (B) To monitor the effectiveness of the chromatographic approach, we ran SDS-PAGE followed by silver staining of the samples at different stages of the purification. Naïve MM indicates a master mix that was not altered in any way. MM eIF4G3Δ-HA indicates master mix after IVTT of eIF4G3Δ-HA. 1st, 2nd and 3rd flow through refer to 3 consecutive washes of the columns. Purified eIF4G3Δ-HA refers to eluted fraction (C) The same gel was then dried and analyzed by autoradiography. (D) The silver stained gel and the autoradiography film were superimposed and shown at higher resolution. The same approach was used for HA-tagged wild-type eIF4G3. (E) Purified products were detected by Western blot analysis at a concentration of 0.1 µg of purified protein per lane. (TIF) [file ppat.1002447.s002.tif]

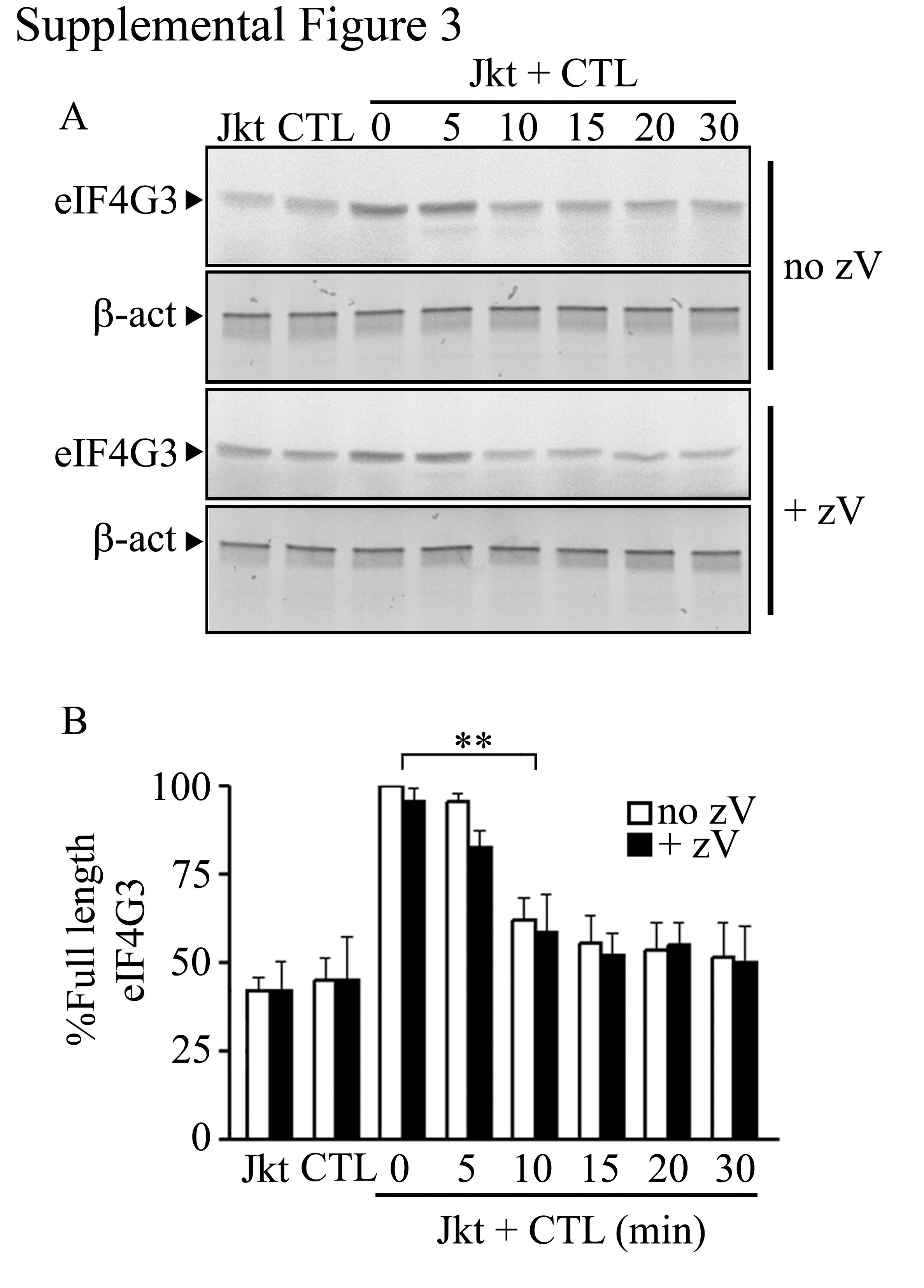

Supplement: Figure S3 — CTL-treatment of Jurkat cells induces eIF4G3 degradation. (A) Expression of eIF4G3 was accessed by Western blot analysis in the first 30 min after treating Jurkat cells with CTL in the absence of zVAD-fmk (no zV) or in its presence (+zV). (B) Densitometric analysis of (A) where the eIF4G3/β-actin (β-act) ratio was calculated and the highest densitometric value was a 100%. The data was plotted as a percentage of the maximum densitometric value. Statistical significance: p<0.01 (**); (n = 6 of 6 independent experiments). (TIF) [file ppat.1002447.s003.tif]

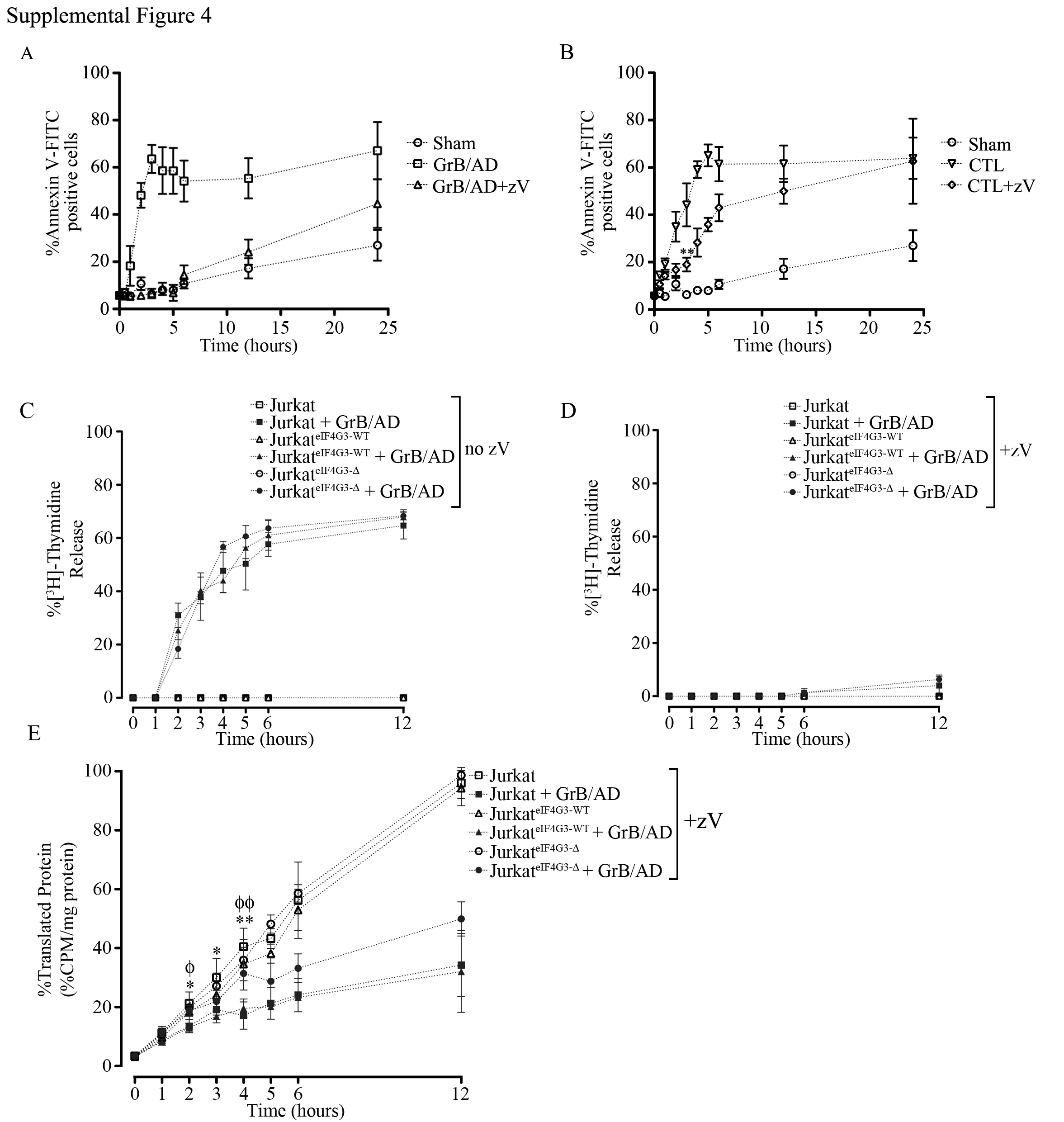

Supplement: Figure S4 — GrB and CTL-induced changes in apoptosis and translation. Jurkat cells were treated with (A) GrB/AD and (B) CTL for 24 hr. %Annexin positive cells were measured at 1 hr intervals up to 6 hr and then at 12 hr and 24 hr. Sham treatment included AD at a concentration of 10 PFU/cell. For (A and B) statistical significance of p<0.01 (**) is shown where * compares statistical significance of Jurkat cells treated with CTL vs. Jurkat cells treated with CTL in the presence of zV; (n = 5 of 5 independent experiments). (C–E) Untreated Jurkat cells, Jurkat cells transfected with wild-type eIF4G3 (JurkateIF4G3-WT) and Jurkat cells transfected with the GrB-resistant eIF4G3 mutant (JurkateIF4G3Δ) were treated with GrB and AD (GrB/AD). (C and D) DNA fragmentation was measured as %[3H]-Thymidine release (C) in the absence of zVAD-fmk (no zV) and (D and E) in the presence of zVAD-fmk (+zV). (E) %Translated protein was measured; Statistical significance: p<0.05 (* and φ) p<0.01 (** and φφ); where * compares statistical significance of JurkateIF4G3-WT+GrB/AD vs. JurkateIF4G3Δ+GrB/AD; and φ compares statistical significance of Jurkat+GrB/AD vs. JurkateIF4G3Δ+GrB/AD; (For C–E, n = 3 of 3 independent experiments). (TIF) [file ppat.1002447.s004.tif]
