# Supplementary material for: Granzyme B Inhibits Vaccinia Virus Production through Proteolytic Cleavage of Eukaryotic Initiation Factor 4 Gamma 3
Source: PLoS Pathog. 2011 Dec 15;7(12):e1002447. doi: 10.1371/journal.ppat.1002447 (PMC3240606; doi:10.1371/journal.ppat.1002447)
Supplement: Table S1 — Sequence alignment for GrB substrate cleavage sites, related to Figure 2. GrB cleaves these substrates between the Aspartate at the P1 and the P1' residue. The 5 residues shown for each substrate (P4 to P1') are critical for GrB catalytic cleavage. The GrB substrates are Caspase-3 (casp-3), BH3 Interacting domain Death agonist (Bid), eukaryotic initiation factor 4 gamma 3 (eIF4G3), and centromeric protein b (CENP-B). (DOC) [file ppat.1002447.s005.doc]

**Supplemental Table 1**

Residue location relative to cleavage site

| **GrB Substrates** | P4 | P3 | P2 | P1 | P1’ |
| --- | --- | --- | --- | --- | --- |
| caspase-3 | I^171^ | E^172^ | T^173^ | D^174^ | S^175^ |
| Bid | I^72^ | E^73^ | A^74^ | D^75^ | S^76^ |
| eIF4G3 | I^1405^ | E^1406^ | S^1407^ | D^1408^ | S^1409^ |
| CENP-B | V^454^ | D^455^ | S^456^ | D^457^ | E^458^ |

**Supp. Tab. 1:** Sequence alignment for GrB substrate cleavage sites, related to Figure 2. GrB cleaves these substrates between the Aspartate at the P1 and the P1’ residue. The 5 residues shown for each substrate (P4 to P1’) are critical for GrB catalytic cleavage. The GrB substrates are Caspase-3 (casp-3), BH3 Interacting domain Death agonist (Bid), eukaryotic initiation factor 4 gamma 3 (eIF4G3), and centromeric protein b (CENP-B).
